# Sampling, identification and sensory evaluation of odors of a newborn baby’s head and amniotic fluid

**DOI:** 10.1038/s41598-019-49137-6

**Published:** 2019-09-04

**Authors:** Tatsuya Uebi, Takahiko Hariyama, Kazunao Suzuki, Naohiro Kanayama, Yoshifumi Nagata, Saho Ayabe-Kanamura, Shihoko Yanase, Yohsuke Ohtsubo, Mamiko Ozaki

**Affiliations:** 1 Department of Biology, Graduate School of Science, Kobe University, Kobe, Japan; 2 Preeminent Medical Photonics Education & Research Center, Institute for NanoSuit Research, Hamamatsu University School of Medicine, Hamamatsu, Japan; 3 Perinatal Medical Center, Hamamatsu University Hospital, Hamamatsu, Japan; 4 Department of Obstetrics and Gynecology, Hamamatsu University School of Medicine, Hamamatsu, Japan; 5 Faculty of Science and Engineering, Iwate University, Morioka, Japan; 6 Division of Psychology, Faculty of Human Sciences, University of Tsukuba, Tsukuba, Japan; 7 Department of Psychology, Graduate School of Humanities, Kobe University, Kobe, Japan

**Keywords:** Chemical ecology, Human behaviour

## Abstract

For baby odor analyses, noninvasive, stress-free sample collection is important. Using a simple method, we succeeded in obtaining fresh odors from the head of five newborn babies. These odors were chemically analyzed by two-dimensional gas chromatography coupled with mass spectrometry (GC × GC-MS), and compared with each other or with the odor of amniotic fluid from the baby’s mother. We identified 31 chemical components of the volatile odors from neonate heads and 21 from amniotic fluid. Although 15 of these components were common to both sources, there was an apparent difference in the GC × GC patterns between the head and amniotic fluid odors, so the neonate head odor might be individually distinct immediately after birth. Therefore, we made artificial mixtures of the major odor components of the neonate head and maternal amniotic fluid, and used psychological tests to examine whether or not these odors could be distinguished from each other. Our data show that the artificial odor of a neonate head could be distinguished from that of amniotic fluid, and that the odors of artificial head odor mixtures could be correctly discriminated for neonates within an hour after birth and at 2 or 3 days of age.

## Introduction

In mammals, odor cues emitted by newborn babies and/or mothers are essential to establish a mother-infant interaction at parturition^1,2^. Soon after birth the infants must initiate milk suckling to survive. In the mouse, maternal odors promote first suckling of neonates^3^. In humans, the sense of smell is arguably the most underestimated sense among our five fundamental senses but is known to have some social communicative functions^4–8^. As with other mammals, human neonates can also perceive environmental odors as cues to recognize aspects of the surrounding world and to seek their mothers. The olfactory system, which already functions in the fetus surrounded by the amniotic fluid, is one of the earliest emerging systems in fetal development^9^. Therefore, the odor of the amniotic fluid is thought to be a memorable odor not only for neonates but also for the fetus. It has been suggested that infants are attracted to or soothed by the odor of the amniotic fluid via olfactory learning during the fetal stage^10,11^ and later acquire various communication skills through reinforcement learning of favorability associated with their mothers^12–14^. They can memorize and be attracted to odors of their mother and mother-related odors such as the odors of milk and amniotic fluid^11–17^. At the same time, mothers and/or other adults may be aware of odors emitted by babies, and actively seek the smell of such odors in daily child-rearing^18,19^.

It has also been reported that human body odors are used as chemical cues for kin recognition^20–23^. Kaitz *et al*. (1987), Porter, Cernoch & McLaughlin, (1983) and Schaal *et al*. (1980) independently investigated the accuracy by which mothers can discriminate between their own baby and unrelated babies by odor cues derived from the babies^24–26^. The major findings of these experiments are consistent: within several days postpartum, mothers reliably identified their own neonate’s t-shirt by odor cues alone when it was presented along with t-shirts worn by other babies. Blindfolded mothers could recognize their offspring accurately when sniffing the heads of three infants, one of whom was their own offspring^27^. Moreover, Schaal and Marlier (1998) showed that both human mothers and fathers can discriminate the odors of two different samples of amniotic fluids, one from their own baby and the other from an unrelated baby^28^. Data suggest that human amniotic fluid carries individualized odor properties, leading to the hypothesis that it plays a role in the initiation of parent-infant interactions. Dubas *et al*. (2009) showed that both mother and father can discriminate between their own child and a control child by smell, and also made a preliminary assessment of whether olfactory discrimination is linked to odor preferences and parental investment^29^. Recently, Croy *et al*. (2017) used a study based on odor ratings to suggest that babies smell wonderful to their parents but teenagers do not^30^.

These earlier studies have highlighted the importance of olfactory cues in the formation and development of mother-infant relationships but (with one or two exceptions^31,32^) there has been no systematic investigation to analyze or identify the essential components of such odors serving as olfactory cues. In contrast, metabolites in human amniotic fluids have been investigated for medical purposes such as disease diagnosis. In almost all cases using gas chromatography with mass spectroscopy (GC-MS) for chemical analyses, amniotic fluid samples were pre-treated to stabilize volatile metabolites, to enable analysis of both volatile and nonvolatile substances together by the GC-MS system^33–35^. However, such analyses using liquid samples confound volatile odor components with nonvolatile water-soluble components.

There are few papers focusing on the chemical analysis of volatile odor components of human neonate heads and amniotic fluid. The focus of the present paper is on an accurate analysis of volatile chemicals in amniotic fluid and also the fresh odors of the newborn baby, both of which are sensed by the olfactory system.

Direct sampling is challenging, both technically and ethically, so in previous research on neonate odors, odor samples have been collected not directly from babies but indirectly from clothes such as t-shirts worn by the babies^36–39^. In the present study, we were able to analyze the volatile substances in fresh odor samples obtained directly from the heads of newborn babies using a stress-free method. However, in view of the delicate nature and ethics of sampling human neonates, sample numbers were limited for this pioneering study.

In addition, we carried out a sensory evaluation study with young adults, using an artificial mixture of 14 major components of volatile substances emitted by the neonate head within an hour of birth, 13 components from the head of babies 2 or 3 days postpartum, and 10 components from amniotic fluid obtained at the time of birth. Considering the psychological and/or physiological effects of the odor of amniotic fluid and neonates, which are probably among the earliest cues to promote mother-infant communication^10,18,19,29,38,39^, the combination of chemical analysis combined with sensory evaluation reported here is a potentially important contribution to research on early mother-infant bonding and communication.

## Results

### Chemical identification of odor components of a baby’s head and the mother’s amniotic fluid

**Figure 1:**
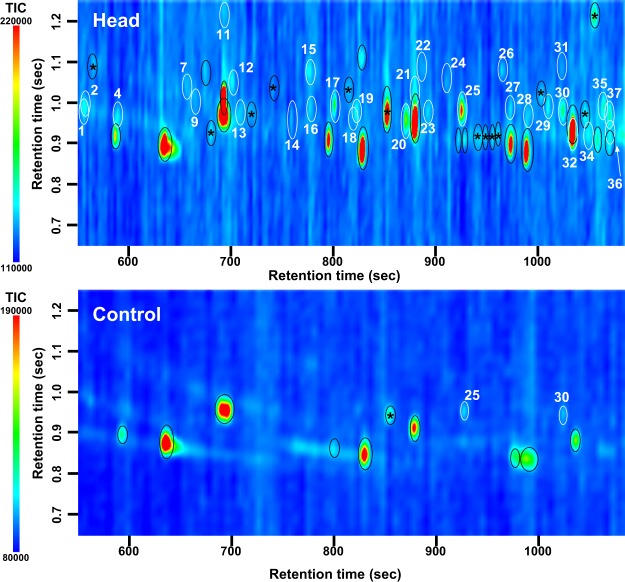
Contour plots of two-dimensional gas chromatograms of the volatile components of odors from a baby’s head (Top) and control (Bottom). GC × GC-MS analysis. Chemically identified spots are encircled. Numbered spots encircled in white correspond to the numbered compounds in Table 1. Nonspecific spots are encircled in black: those with asterisks are solvent- or synthetic resin-related material contamination; those without asterisks indicate siloxanes and/or their derivatives from the adsorbent beads.

**Figure 2:**
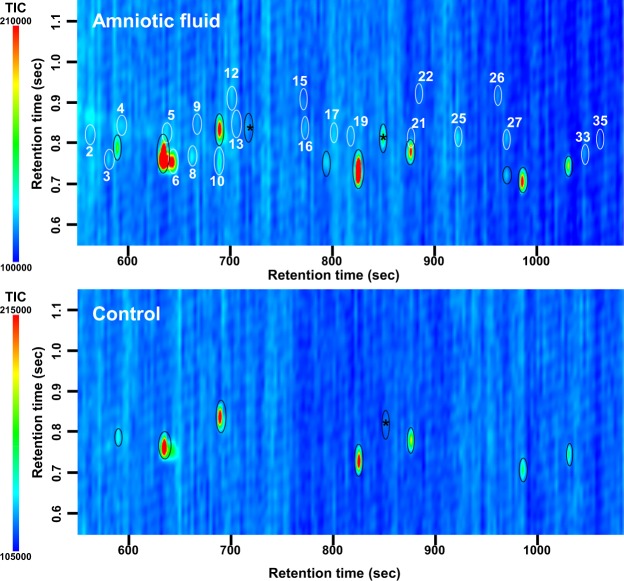
Contour plots of two-dimensional gas chromatograms of the volatile fraction of odors from amniotic fluid (Top) and control (Bottom). Labelling conventions as for Fig. 1.

Odor samples were analyzed by GC × GC-MS, enabling discrimination of different spots which were superimposed in one-dimensional GC-MS. Figures 1 and 2 show representative contour plots as two-dimensional (2D) gas chromatograms for the head odor of a baby (Baby 1 in Table 1) and for the amniotic fluid odor of his mother (Mom 1 in Table 1). Here, the results of GC × GC-MS analyses focus on the volatile components of the odors. The contour plots for the baby’s head odor show inseparable spots of compounds of relatively longer retention times, indicating longer retention times than the spots shown in Fig. 1. Such inseparable spots mainly include unidentified hydrocarbons or their derivatives, which were presumed to be non-volatile for their retention times. In contrast, there should be no non-volatile substances present on the contour plots for the amniotic fluid odor because these samples were collected by the *in-vitro* headspace sampling method at room temperature and there was no direct contact with the amniotic fluid. Considering these conditions, the 2D gas chromatograms were naturally divided into volatile and nonvolatile parts, and data in Fig. 1 were used only from the volatile part (510–1080 s retention time).

**Table 1:** Tentative chemical identification of odors from the head of five sampled babies and amniotic fluid from two mothers.

No	Compound	Head retention time* 1D, 2D (sec)	Amniotic fluid retention time^†^ 1D, 2D (sec)	Measured mass	Area ^§^						
					Head of baby 1	Head of baby 2	Head of baby 3	Head of baby 4	Head of baby 5	Amniotic fluid of mom 1	Amniotic fluid of mom 2
1	2,3-Butanediol	555, 0.952		45	13857	9513	6481	3119	6027	nd	nd
2	2-Butenoic acid	555, 0.980	560, 0.804	86	4958	2597	5328	2595	4760	2119	1130
3	4-Methylheptane		578, 0.759	43	nd	nd	nd	nd	nd	10244	38785
4	2,4-Pentadienal/Hexanal	588, 0.980	590, 0.801	56	5831	9376	9780	6163	9500	2142	1538
5	4-Methyl-1-pentanol		635, 0.832	56	nd	nd	nd	nd	nd	19220	20735
6	2,4-Dimethylheptane		641, 0.746	43	nd	nd	nd	nd	nd	69064	85678
7	1,3-Butandiol	651, 1.050		57	2654	675	734	729	874	nd	nd
8	2,4-Dimethyl-1-heptene		659, 0.766	43	nd	nd	nd	nd	nd	24576	24595
9	Valeric acid	666, 0.990	665, 0.840	60	2448	1539	4640	3453	8261	1338	3315
10	3-Ethylhexane		686, 0.752	43	nd	nd	nd	nd	nd	40468	41401
11	2(5H)-Furanone	690, 1.220		55	21017	10076	15656	15420	12497	nd	nd
12	Cyclohexanone	699, 1.050	698, 0.917	55	39013	8431	11748	8630	2597	17754	13550
13	Heptanal	705, 0.990	704, 0.834	41	9599	8350	12107	3906	8024	1805	1530
14	sec-Butyl formate	756, 0.960		45	10544	978	3249	2722	5476	nd	nd
15	Benzaldehyde	771, 1.090	770, 0.911	77	23384	13226	18305	14161	12601	1039	2017
16	Hexanoic acid	774, 0.990	770, 0.838	60	9732	4637	62552	49043	52047	19536	39541
17	6-Methyl-5-hepten-2-one	798, 1.000	794, 0.834	43	70127	12871	30348	29145	25870	2406	1183
18	2-Pentylfuran	816, 0.960		81	3325	3940	8723	5063	3399	nd	nd
19	Octanal	819, 0.980	815, 0.825	41	15689	3586	14925	8274	6408	1496	449
20	Limonene	867, 0.960		68	56309	539	312	468	153	nd	nd
21	Heptanoic acid	876, 0.982	871, 0.825	41	tr	tr	18473	12855	10612	6079	8140
22	1-Phenylethanone	885, 1.090	880, 0.924	120	3048	2656	2734	3387	1938	516	339
23	1-Octanol	888, 0.970		41	10433	3044	3548	2805	4204	nd	nd
24	2-Phenyl-2-propanol	906, 1.050		43	17303	9195	11873	9590	8444	nd	nd
25	Nonanal	921, 0.980	916, 0.812	57	67397	44880	69504	57575	96046	10461	18350
26	Benzoic acid	960, 1.080	955, 0.902	105	8951	5034	12470	10199	10022	2466	3222
27	Octanoic acid	969, 0.990	964, 0.818	60	13438	5575	68835	41415	29371	15333	27526
28	2-Octen-1-ol	987, 0.980		57	1506	597	1444	3950	1200	nd	nd
29	Menthol	1005, 1.000		41	13674	2142	13649	10392	8543	nd	nd
30	Decanal	1020, 0.980		41	24435	5282	16089	20972	19465	nd	nd
31	Azulene	1020, 1.090		128	11786	7002	11600	8737	8308	nd	nd
32	Dodecane	1029, 0.930		57	82702	17841	106125	62592	39950	nd	nd
33	2-Methyl-2-decanol		1039, 0.779	59	nd	nd	nd	nd	nd	7325	4340
34	4-Methyldecane	1044, 0.910		57	13901	2150	21599	14563	5230	nd	nd
35	Nonanoic acid	1059, 0.990	1054, 0.805	60	12279	3564	48268	38110	25391	10835	15878
36	2-Propyl-1-heptanol	1065, 0.920		57	3695	1775	3309	1523	2070	nd	nd
37	3-Hexyn-1-ol	1065, 0.970		68	18294	7997	21246	13219	13188	nd	nd

*Retention time in Baby 1; ^†^Retention time in Mom 1; ^§^Corrected value of area for the control.

There were 31 specific spots of volatile odor compounds obtained from the head of babies (Fig. 1, Top) and 21 from the amniotic fluid (Fig. 2, Top). In addition to the specific volatile odor compounds (white circles), siloxanes and their derivatives were detected. These are likely to be derived from the adsorbent beads (black circles without asterisks in Figs 1 and 2), or nonspecific contaminants derived from solvent- or synthetic resin-related materials (black circles with asterisks). The odors of both a newborn baby’s head (Fig. 1, Top) and amniotic fluid (Fig. 2, Top) contained 15 specific, commonly detected components, but the GC × GC pattern of volatile odors is apparently different between them. The controls (Figs 1 and 2, Bottom) indicate similar GC × GC patterns except for two spots of nonanal and decanal in Fig. 1, both of which are much weaker in the control (Fig. 1, Bottom) than the sample pattern (Fig. 1, Top). Siloxanes and their derivatives also are seen in both control patterns of Figs 1 and 2 (black circles without asterisks), although the control pattern of Fig. 1 has two such spots more than that of Fig. 2 (reason unknown).

From the total analytical results aggregated for all tested samples (five babies’ heads and two amniotic fluid samples), the 37 volatile odor components obtained are listed in Table 1. This table shows the area of well-separated m/z, which is corrected by subtracting the area of the corresponding peak if it appeared in the control. Table 1 shows that, except for limonene, volatile odor components from the baby’s head were commonly detected in all five babies tested, regardless of their odor sampling time after birth. They included large amounts of nonanal and other aldehydes (heptanal and octanal), and carboxylic acids (valeric, hexanoic, heptanoic, octanoic and nonanoic acids). The quantity of carboxylic acids gradually increased after birth. In particular, the amount of heptanoic acid was at the control level in Babies 1 and 2 (collected within an hour after birth), but markedly large amounts were detected in Babies 3–5 (sampled 2 or 3 days postpartum). In addition, several hydrocarbons were detected in the odors from both the baby’s head and the amniotic fluid.

### Difference in the GC × GC pattern among odors of babies’ heads and amniotic fluids

**Figure 3:**
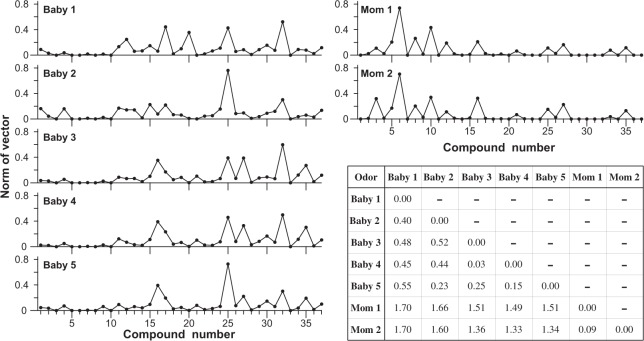
Variation of norm of odor composition from a baby’s head and from amniotic fluid. For explanation of the identification of babies and moms, see text. Inset indicates square-difference between pairs of odors.

Figure 3 shows the 37 identified odor compounds in the babies’ heads and amniotic fluids (compounds no. 1 to 37 in Table 1) plotted as norms in a 37-dimensional vector space including each odor sampled. The patterns show apparent differences in odor composition, with those obtained from a baby’s head (Babies 1–5) indicating relatively smaller amounts in substances of shorter retention times, while amniotic fluid (Moms 1 and 2) had larger amounts of substances of longer retention times. The samples of odors from the baby heads differ among each other more than do those obtained from amniotic fluid. The odor profiles of Babies 1 and 2, which were collected within an hour after birth, look less similar to each other than those of Babies 3–5 collected 2 or 3 days after birth.

The inset of Fig. 3 shows quantitative squared-differences in GC × GC pattern among the odor samples, with larger and smaller values suggesting correspondingly larger and smaller degrees of difference between pairs of odors. The head odor profile difference between Babies 1 and 2 (squared-difference = 0.40) was larger than that among Babies 3–5 (squared-differences = 0.03, 0.15 or 0.25). This difference was also similar to the difference between Baby 1 or 2 and Baby 3, 4 or 5 (squared-difference = 0.44 to 0.55) except between Babies 2 and 5 (squared-difference = 0.23). Although Moms 1 and 2 were the real mothers of Babies 1 and 2, respectively, the amniotic fluid odor difference between Moms 1 and 2 (squared-difference = 0.09) was much smaller than the head odor difference between Babies 1 and 2 (squared-difference = 0.40). The odor difference between the amniotic fluid of Moms 1 or 2 and the head of each of the babies (squared-difference = 1.33 to 1.70) was quite large regardless of the combination for mother-baby comparison.

The amniotic fluid odor contains some volatile hydrocarbons that were not detected in the baby’s head odors: 4-methylheptane, 2,4-dimethylheptane, and 2,4-dimethyl-1-heptene (Table 1). There were 15 components commonly detected both in the odors of a baby’s head and in the amniotic fluid, including 3 aldehydes (heptanal, octanal and nonanal) and 5 carboxylic acids (valeric, hexanoic, heptanoic, octanoic and nonanoic acids). The odors of Babies 1 and 2 contain carboxylic acids, including hexanoic, heptanoic, octanoic and nonanoic acids. The carboxylic acid content of the head odor of Baby 1 and Baby 2 was less than for that of Baby 3, 4 or 5 or the amniotic fluid odors of Moms 1 and 2.

### Sensory evaluation of volatile odors from a baby’s head and from amniotic fluid

**Figure 4:**
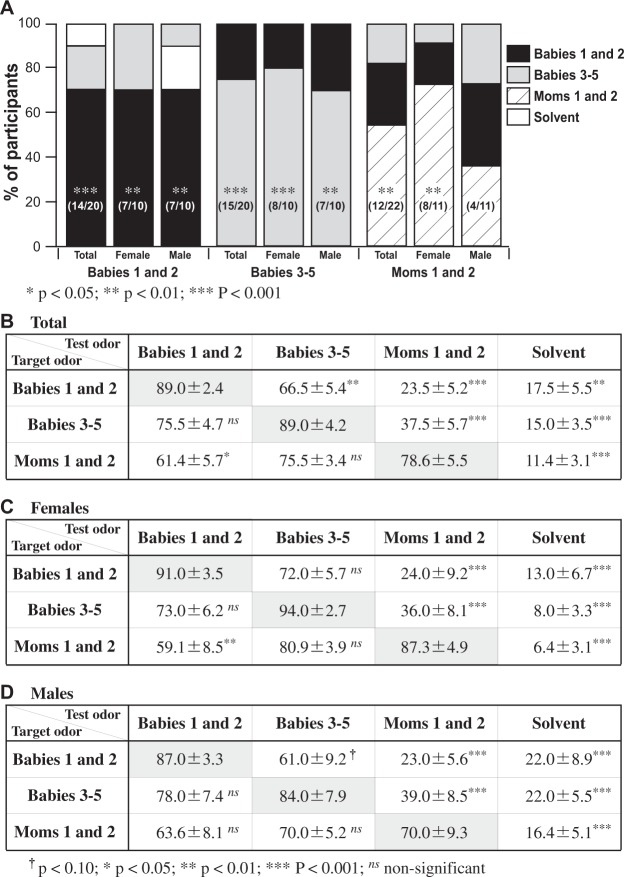
Sensory evaluation of artificial odor mixtures mimicking mean odor composition of a baby’s head, immediately postpartum and a few days after birth, and the amniotic fluid. (**A**) Percentage of participating evaluators who chose each of the four test odors, as a function of target odor (Babies 1 and 2; Babies 3–5; Moms 1 and 2) and participant type (total, female, male). For each target odor, the correct choice rate (no. of participants choosing the correct identification as % of total relevant participants) is indicated at the bottom of the bar. (**B**–**D**) Mean similarity ratings (% ± SD) between the target odor and each of the four test odors, based on (**B**) the total number of evaluators (n = 62), (**C**) female evaluators only (n = 31), and **(**D) male evaluators only (n = 31). The similarity ratings for the same target-test combination are in gray boxes. Asterisks indicate a significant difference from the similarity rating of the matched stimuli (see key below figure; chance level 25%).

The evaluation experiment used three target odor samples (i.e., mixtures based on the mean odor components of the heads of Babies 1 and 2, heads of Babies 3–5, and amniotic fluids of Moms 1 and 2; see Supplemental Tables 1–3), and four test odors (the same as the target odor mixtures, plus odor-free solvent control). Participants were presented with one of the three target odors, and then provided with the four test odors. When the target odor was either the head odor mixture based on Babies 1 and 2 or that of Babies 3–5, the correct identification rate for all participants was above 70% (significantly different from the chance level of 25% by a binomial test; p < 0.05, p < 0.01 and p < 0.001 are indicated by *, **, and ***, respectively). The correct identification rates were also above 70% when data were analyzed separately for male and female participants. For amniotic fluid, the total correct identification rate was lower (55%) than for babies’ head odors for all participants but still significantly greater than chance. The performance by female participants was much better (73%) than by males (36%), whose performance was not significantly greater than the chance level (Fig. 4A).

These results were corroborated by a series of repeated-measures analysis of variance with the test odor sample as the independent variable. The repeated-measures ANOVAs were conducted separately for the three target conditions. As shown in Fig. 4B, the highest similarity ratings were associated with the matched-odor condition (i.e., the test odor sample was the same as the target odor sample). The main effect of test odor type was significant for the three analyses: F(3, 76) = 50.70, p < 0.001 for the Babies 1 and 2 condition; F(3, 76) = 54.51, p < 0.001 for the Babies 3–5 condition; F(3, 84) = 46.06, p < 0.001 for the amniotic fluid condition. A series of post-hoc tests with Tukey’s method indicated that the matched-odor similarity rating was significantly different from the unmatched-odor similarity ratings. When the target odor was “Babies 1 and 2,” participants perceived it as significantly more similar to the matched test odor of “Babies 1 and 2” (89.0%) than unmatched test odors (66.5% for Babies 3–5; 23.5% for Moms 1 and 2; 17.5% for solvent alone). The same pattern held for the other target odors, but with some exceptions. When the target odor was “Babies 3–5,” participants perceived it as significantly more similar to the matched test odor of “Babies 3–5” (89.0%) than the two unmatched test odors (37.5% for Moms 1 and 2; 15.0% for Solvent). However, its similarity to the unmatched test odor of “Babies 1 and 2” (75.5%) was not significantly different from the matched similarity (89.0%). When the target was “Moms 1 and 2,” participants perceived it as significantly more similar to the matched target of “Moms 1 and 2” (78.6%) than the two unmatched targets (61.4% for Babies 1 and 2; 11.4% for solvent alone). However, target similarity to the unmatched test odor of “Babies 3–5” (75.5%) was not significantly different from the matched similarity (78.6%). These results indicate that participants generally perceived the three target odors to be more similar to the matched test odors than the unmatched test odors.

We also conducted comparable repeated ANOVAs for each sex separately (see Fig. 4C,D). The results were mostly comparable with the analyses involving both sexes (Fig. 4B). However, probably due to the small sample size, a few additional comparisons that were significant in the main analyses (Fig. 4B) failed to reach the level of statistical significance. It is also noteworthy that the perceived similarity ratings between babies’ head odors (either Babies 1 and 2 or Babies 3–5 as target) and amniotic fluid odor (test) were relatively low (23.5% for target Babies 1 and 2; 37.5% for target Babies 3–5 in Fig. 4B). Nonetheless, the perceived similarity ratings between amniotic fluid odor (target) and babies head odors were modest (61.4% for test Babies 1 and 2; and 75.5% for test Babies 3–5 in Fig. 4B). Thus, asymmetric perceptions of similarity for amniotic fluid odor were notable in the present olfactory evaluation.

In spite of some unexpected patterns, then, on the whole the student evaluators were shown to be capable of discriminating the three types of odor samples from each other and from the control odor of solvent alone.

## Discussion

Chemical analyses of natural odors have been performed widely, and our understanding of volatile organic chemicals (VOCs) derived from various odor sources in natural environments, houses, foods, or even human bodies has greatly advanced^40–42^. Recent progress has relied much on methodological developments, such as the solid-phase micro-extraction (SPME) method combined with mass-spectrometry. Indeed, SPME is a useful odor adsorbing tool, but not applicable to odor sources as delicate as those of newborn babies. Even newborn babies try to avoid unpleasant treatments, and this renders it difficult to collect odors from neonates under stress-free conditions. It is for these reasons that few observations are available on the odors of newborn babies, and those that have been done have typically made use of the clothes used by babies instead of collecting the odors directly^36–39^.

In the present study, we successfully collected fresh odors of newborn babies immediately after birth by using MonoTrap silica beads. During odor sampling, no baby cried or fretted but remained rather relaxed or even sleeping in the bed or its mother’s arms. Thus, we conclude that our odor sampling method was successfully applicable even to newborn babies soon after birth.

Odor compounds such as those emitted from the human body, tend to change upon oxidization^43^. Indeed, as shown in Fig. 3, the head odors of babies 2 or 3 days after birth (Babies 3–5) tended to differ from those sampled within an hour after birth (Babies 1 and 2). It is noted, however, that without more extensive sampling it is difficult to differentiate the effects of odor changes attributable to oxidization from random differences among individual babies. Nevertheless, it was observed that the odor of the baby’s head, once trapped in the monolithic silica adsorbent, hardly changed in its GC × GC pattern following storage overnight in a refrigerator (unreported observations). A disadvantage of this method is the inevitable contamination of the odor samples by siloxanes and their derivatives because the MonoTrap beads are composed of monolithic silica. The GC data also included nonspecific peaks concerned with solvent- or synthetic resin-related contaminants, which were probably derived from the use of surgical gloves, etc. Hence, analysis of odor samples by GC × GC-MS (rather than GC-MS alone) is recommended to increase the resolution for detecting specific spots which may otherwise be hidden among those of contaminants.

Amniotic fluid provides the total environment for a fetus. Odors derived either from amniotic fluid or the body surface of the fetus can therefore be expected to be present at their interface. Thus, the odor on the body surface of a fetus and probably of a newborn baby soon after birth can be expected to have common components, at least in part, common with those of the amniotic fluid. However, the body surface of a newborn baby and the amniotic fluid are expected to contribute their own specific-odor components. As shown in Table 1, odor components specific to the baby’s head or the amniotic fluid presumably cause the characteristic differences in the GC × GC pattern (compare between Figs 1 and 2). The odor composition of neonates shows dissimilarities to those of both amniotic fluid and babies 2 or 3 days postpartum (Fig. 3). This suggests that the head odor of a newborn baby is not composed only of odor compounds lingering from the amniotic fluid. Also, Babies 3–5 had 14 compounds in common with Babies 1 and 2, suggesting that babies continuously secrete these 14 odor compounds, which are therefore detected even after the baby has been bathed several times.

As for the change of the odor composition of a baby’s head, aldehydes were maintained at mostly the same level, but carboxylic acids increased three-fold in the 2 or 3 days after birth. For example, nonanal was the most abundant odor component both in the neonate (Babies 1 and 2; average occupancy of peak area = 27%) and Babies 3–5 (2 or 3 days postpartum; average occupancy of peak area = 29%); while nonanoic acid increased five-fold during the few days after birth, which might be explained by oxidation of aldehydes following exposure of the neonate to air instead of the amniotic fluid. Although the odor compounds specific to the amniotic fluid were not likely to be derived from the baby’s body surface, some odor compounds might be transferred from the fetus to the amniotic fluid in utero. Generally, carboxylic acids are more easily dissolved into an aquatic environment than aldehydes, so those released from the fetus would be dissolved in the amniotic fluid. Thus, either one of both factors (transfer of carboxylic acids from fetus to the amniotic fluid; and oxidization of aldehydes after birth) might explain the quantitative change of carboxylic acids from Babies 1 and 2 to Babies 3–5 (see Table 1), which is reflected in the difference in GC × GC patterns between them (Figs 1–3).

It has been reported that body odors are used as olfactory cues for kin recognition. Many studies have suggested that the relationship between parents and their own babies attracting each other is based on mutual detection of odors specific to the other^18,20–22,28,29^. Even small differences in some specific components or their mixture pattern are possibly important for kin recognition of newborn babies by their parents, but this would require highly sensitive olfactory capability. In the present study, we found that the volatile component of odors from the heads of Babies 1 and 2 immediately after birth could reflect individual odor profiles. After that, the volatile odor composition changed, so that individual difference tended to decrease in a few days. Data in Fig. 3 show smaller odor differences among Babies 3–5 than that between Babies 1 and 2, suggesting that a baby can more strongly express its individuality soon after birth rather than a few days later. However, if newborn babies stay in a common nursery in a hospital with a standard environment, their body odors might tend to become uniform. This might explain why a larger odor difference occurred among newborn babies immediately after birth (square difference 0.40 in Fig. 3) rather than 2 or 3 days after birth (0.03–0.25). Figure 3 indicates that the square-difference between the head odors of Babies 1 and 2 (0.40) is within a similar range to that between any two head odors of Babies 1–5 (0.23–0.55). However, Fig. 4 suggests that the artificial mean odor mixture of Babies 1 and 2 can be discriminated from the artificial mean odor mixture of Babies 3–5. This suggests that two odor mixtures with a squared-difference in the range 0.23–0.55 can be discriminated, and therefore this would apply to the odors of Babies 1 and 2, the squared-difference between which is 0.40. At this stage, however, further discussion on baby odor individuality (or its role in kin recognition) is precluded in view of the relatively few odor samples from newborn babies available in the present study.

Not only kin recognition but also early mother-infant bonding and communication may be mediated by odors emitted from infants^25–27,38^. As humans are social species, infants might rely on attracting the attention of their mother, father, other relatives or other adults in situations where their survival is endangered. Considering the helplessness of the newborn human baby and the necessity of protection not only by its mother but also by family, community and society, it might be reasonable to expect that a newborn baby’s odor, even immediately after birth, could attract the attention of adults of various statuses. Thus, it may be worth investigating the sensory evaluation of infant odors by adults other than parents. The sensory evaluation experiment reported here was performed with young adults aged 18–24 y but they received no explanation about the odor, so this experiment was not keenly directed to mother-infant bonding responses or kin recognition. The results suggest that both female and male adults can readily distinguish the odor of a neonate’s head from that of a baby 2–3 days old (Fig. 4). This could be consistently explained by the chemical analyses of differences in the natural head odor among babies (Fig. 3).

It was interesting that female evaluators showed a higher rate of correct identifications than males for the amniotic fluid odor (Fig. 4A). There have been many reports suggesting that the female olfactory sense is better than that of males, a conviction that has achieved the status of an urban legend^44^. However, in olfactory discrimination tests where the participants are requested to decide which of the two or three odors is different, the data obtained are not consistent in terms of potential sex differences^45,46^, as seen also for the sensory evaluation tests reported in the present study (except for that on the amniotic fluid odor). Thus, there is presumably some reasonable explanation for this exceptional intersexual difference, but it is obscure at the present time. On the odor similarity of amniotic fluid to the baby’s head and of the baby’s head to the amniotic fluid, the answers obtained were asymmetric (Fig. 4B). This might be the result of some unknown psychological effect concerned, for example, with the presentation order of odor samples.

Needless to say, newborn babies are limited in their capacity for meaningful communication. However, they can cry and also emit their own odors even immediately after birth. Acoustic stimulation with the cry of a baby emotionally influences mothers, and it also seems very likely that the baby odors may have a psychological effect on mothers^18,29^. Okamoto *et al*. (2016) have noted that experience of detecting a baby’s odor by the mother induces feelings of care and attention directed towards her baby^19^. When a mother is detecting her baby’s odor, her brain activity is significantly increased in the bilateral prefrontal cortex^39^. Using fMRI, maternal status-dependent activity was found in the thalamus when research participants were exposed to the body odor of a newborn baby^37^. However, in all such cases, the odor components or odor pattern emitted by the baby that effectively induce such brain activity have yet to be identified.

For physiological or any other studies to progress, it is stressed here that noninvasive, stress-free collection and convincing analyses of a newborn baby’s odor are indispensable. Our chemical identification relied on referring to the NIST (National Institute of Standard and Technology) library, and on retention index searching for every odorant peak with standard compound peaks. It is also required to mimic the natural odor of a newborn baby precisely by experimenting with artificial odor mixtures. The present study came very close to concocting artificial odors mimicking natural odors, falling a little short of perfection because of a small number of unavailable or unidentified components (Supplemental Tables S1–S3).

Further refinements to the protocol in future studies will include confirmation of odor evaluator marital status, and information on previous experience of interaction with babies, timing of the menstrual cycle, etc^47,48^. These shortcomings preclude further fruitful discussion in the present paper. Nevertheless, there is useful consistency in the experimental data of the chemical analysis and sensory evaluation reported here. In future, if odor sampling from newborn babies could be conducted systematically, sample numbers could be optimized to include variables such as age of sampled babies for various days after birth, separate sampling of both sexes, and modes of delivery by natural birth or caesarean section, to more precisely investigate odor changes with age and other aspects such as the social effects of odors on kin-recognition.

## Methods

### Donors

Sample donors were 5 newborn babies delivered between August 18 and 21, 2017, in the hospital of Hamamatsu University School of Medicine. The participant mothers were assumed to have typical Japanese diets at home, although we obtained no individual information about food habits. These donor babies and mothers, who are native Japanese, were individually identified and in this study are referred to as Babies 1–5 and Moms 1 and 2. Baby 1 is the child of Mom 1 and Baby 2 the child of Mom 2. Babies 1 (male), 3 (female), 4 (male) and 5 (male) were delivered by the normal vaginal route, while Baby 2 (female) was delivered by caesarean section. The odor samples of Babies 1 and 2 were collected within 1 hour of birth. The body was carefully and gently wiped with clean towels before odor sampling. The odor samples of Babies 3 and 4 were collected 2 days after birth, and of Baby 5 collected 3 days after birth. The babies were bathed every day. Odor sampling was approved by the institutional review board of Hamamatsu University School of Medicine. We obtained informed consent from all participant mothers. All experiments were performed in accordance with the relevant guidelines and regulations of Hamamatsu University School of Medicine.

### Odor sampling

**Figure 5:**
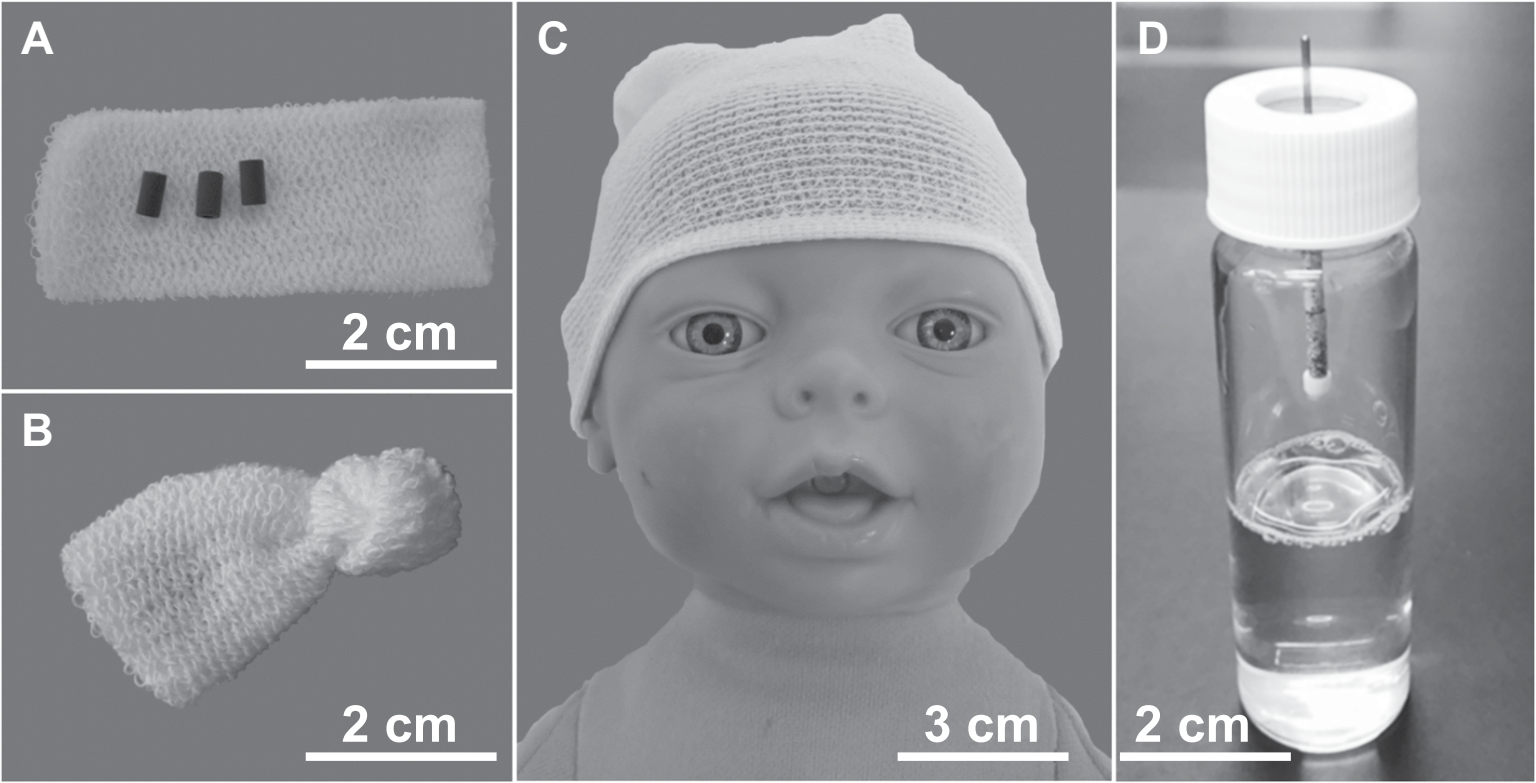
Odor sampling. (**A**–**C**) Odor sampling from a newborn baby’s head. MonoTrap beads (**A**) were held close to the baby’s head by placing them inside a small polyester/cotton elastic net bandage (**B**) mounted gently on the baby’s head (**C**). (**D**) Sampling of the odor of amniotic fluid using MonoTrap beads suspended in the atmospheric space above the fluid contained in a glass jar.

Odor sampling was performed with MonoTrap beads (RGPS TD; GL Sciences Inc., Tokyo Japan), which are composed of a monolithic silica adsorbent readily molded into cylindrical beads. These beads, when placed gently on a baby’s head inside a net bandage (Fig. 5), were totally ignored by the babies. For odor sampling from the baby’s head, five MonoTrap beads were wrapped with a small elastic net bandage (Shinsei Co., Ltd., Nara Japan; Fig. 5A,B) and placed as a lightly-fitting hat on the baby’s head (Fig. 5C). At the same time, we prepared the control samples by wrapping another five MonoTrap beads with a small elastic net bandage and placing them on a clean tissue paper (KimWipe; Nippon Paper Crecia Co., Ltd., Tokyo, Japan) for 20 min at room temperature. Manipulation during these steps was with the use of clean surgical gloves. After 20 minutes, the MonoTrap beads were transferred to 1.5 ml sample vials (Shimadzu Co., Kyoto, Japan), which were kept at 4 °C until subjected to the GC × GC-MS analysis.

For odor sampling from the amniotic fluid, immediately after delivery about 40 ml of amniotic fluid was collected by the attending obstetrician during delivery, 10 ml of which were placed in a glass bottle (40 ml, 22 mm diameter, 80 mm height GL Sciences Inc., Tokyo, Japan). Four MonoTrap beads were suspended from a stainless-steel shaft suspended from the septum in the lid of the bottle and were retained in the headspace of the glass bottle for 2 hours at room temperature (Fig. 5D). For the control odor sampling, another four MonoTrap beads were treated in the same way as for odor sampling from the amniotic fluid, but substituting distilled water for amniotic fluid. The beads were then kept in the sample vials at 4 °C until subjected to the GC × GC-MS analysis.

All preparations, such as wrapping the MonoTrap beads or setting them in the headspace for odor sampling, were done using surgical gloves in a clean, gently ventilated room with restricted entry under stable conditions of 20 °C and 30~40% relative humidity.

### GC × GC-MS analysis

The GC × GC-MS analyses were conducted using a Pegasus 4D GC × GC-TOFMS system (Leco Co., Saint Joseph, MI, USA). A Handy TD portable thermal desorption system (TD265; GL Sciences Inc., Tokyo, Japan) was used to desorb sample odors adsorbed into the MonoTrap beads. One Monotrap bead was used for each analysis. The temperature program for thermal desorption was 40 °C for 0.1 min to 300 °C at 45 °C/min (final hold for 5 min) with a pre-desorption pressure of 180 kPa. Volatiles were transferred to a GC capillary column for separation at an inlet temperature of 250 °C. The primary column was a DB-1 MS column (30 m length × 250 µm i.d. × 0.25 µm film thickness; Agilent Technologies Inc., Santa Clara, CA, USA) and the secondary column was an InertCap 17 column (1.3 m length × 180 µm i.d. × 0.18 µm film thickness; GL Sciences Inc., Tokyo, Japan). Helium was used as carrier gas at a constant flow of 1.5 ml/min. The temperature program for the primary GC oven was 40 °C for 1 min, to 260 °C at 10 °C/min (final hold for 10 min). For the secondary GC oven, the temperature program was 50 °C for 1 min, to 270 °C at 10 °C/min (final hold for 10 min). The modulation period, hot-pulse duration, and cool time between stages were 3.0, 0.65, and 0.85 s, respectively. The transfer line to the Time-of Flight Mass Spectrometer (TOF-MS) detector was operated at 280 °C. The source temperature was 230 °C with a filament bias voltage of -70 eV. The data acquisition rate was 100 Hz in the mass range of m/z 35–500. Data were processed and visualized on 2D chromatograms using Leco Chroma TOF software (Leco Co., Saint Joseph, MI, USA). Individual components were first tentatively identified by referring to the NIST (National Institute of Standard and Technology) library, and more accurately identified by retention index searching with known standard substances, except for 8 of the 37 detected substances (Nos. 4, 10, 14, 28, 33, 34, 36 and 37 in Table 1) for which known standard chemicals were unavailable and/or retention indexes were not consistently determined.

### Data analysis of difference among GC × GC patterns

Differences among the obtained GC × GC patterns were evaluated qualitatively, using the squared Euclidean distance between any two vectors as a criterion difference among the seven vectors obtained from the seven GC × GC patterns described in GC × GC-MS analysis. Each vector consists of 37 components chosen from its original pattern and is normalized to unit size prior to distance calculation. The number of vector dimensions is smaller than that of the original pattern because nonspecific contaminants (i.e. siloxanes, siloxane derivatives and solvent- or synthetic resin-related materials) were excluded from the analysis. The calculated distance ranges from 0.0 to 2.0 because the vectors have non-negative values in this analysis. The distance 0.0 indicates that the two vectors are identical; the value 2.0 indicates that the vectors are orthogonal.

### Artificial odor mixing

Based on the 15 major odor components from the heads of Babies 1 and 2 and of Babies 3–5, and those from the amniotic fluid of Moms 1 and 2, their mean amounts were calculated and artificial odor mixtures to our specifications were obtained upon request to San-Ei Gen F.F.I., Inc. (Osaka, Japan). Supplemental Tables S1–S3 show the composition lists of each artificial mixture (see table captions for factors precluding the achievement of exact duplication). Sensory evaluation tests were conducted using these artificial mixtures diluted (by volume) to 1% by addition of 80% propylene glycol and 19% ethanol, as advised by San-Ei Gen F.F.I., Inc. (Osaka, Japan).

### Sensory evaluation experiment

Sensory evaluations were conducted with 62 participants aged 18–24 years (31 male and 31 female students of Kobe University). The participants were assumed to be unmarried and not raising their own children (a reasonable assumption for this demographic group in Japan: e.g., in a previous study by one of the authors, none of 300 Japanese university/college students were married and/or had a child). The experiments were conducted in small group sessions (5 sessions with 10 participants, 1 session with 9, and 1 session with 3). All experiments were conducted on a blind basis, such that participants were unaware of the type or origin of the odor samples.

Participants were first presented with one of three kinds of artificial odor mixture (target odors) mimicking (1) the head odor of a neonate within 1 h postpartum (Supplemental Table 1); (2) the head odor of a baby 2 or 3 days postpartum (Supplemental Table 2); or (3) odor of amniotic fluid sampled at parturition (Supplemental Table 3). These target odors were impregnated into the tip of filter-paper strips (6 mm × 150 mm). The three artificial odor mixtures and solvent alone were presented to the participants 15 min. later and they were then asked to determine which of the four test samples was identical to the target odor they had smelled earlier. Participants were also required to rate their level of confidence in their decision and each odor sample’s similarity to the target sample, with perceived similarity rated on an 11- point scale at 10% intervals (0% = “not at all similar” to 100% = “very similar”). Each session, rather than each individual participant, was the unit of odor assignment (two sessions each were assigned to each of the three target odor samples). This experiment was approved by the Human Research Ethics Committee of the Graduate School of Humanities, Kobe University. All experiments were performed in accordance with the relevant guidelines and regulations of Kobe University.

## Supplementary information

Supplemental table

## Data Availability

The datasets analyzed during the current study are available from the corresponding author on reasonable requests.
