# Supplementary material for: Sampling, identification and sensory evaluation of odors of a newborn baby’s head and amniotic fluid
Source: Sci Rep. 2019 Sep 4;9:12759. doi: 10.1038/s41598-019-49137-6 (PMC6726625; doi:10.1038/s41598-019-49137-6)
Supplement: Supplementary file 1 — Supplemental table [file 41598_2019_49137_MOESM1_ESM.pdf]

Sampling, identification and sensory evaluation of odors of a newborn baby's head and amniotic fluid.

Tatsuya Uebi[1*], Takahiko Hariyama[2], Kazunao Suzuki[3], Naohiro Kanayama[4], Yoshifumi Nagata[5], Saho Ayabe-Kanamura[6], Shihoko Yanase[1], Yohsuke Ohtsubo[7], Mamiko Ozaki[1]

[1] Department of Biology, Graduate School of Science, Kobe University, Kobe, Japan

[2] Preeminent Medical Photonics Education & Research Center, Institute for NanoSuit Research, Hamamatsu University School of Medicine, Hamamatsu, Japan

[3] Perinatal Medical Center, Hamamatsu University Hospital, Hamamatsu, Japan

[4] Department of Obstetrics and Gynecology, Hamamatsu University School of Medicine, Hamamatsu, Japan

[5] Faculty of Science and Engineering, Iwate University, Morioka, Japan

[6] Division of Psychology, Faculty of Human Sciences, University of Tsukuba, Tsukuba, Japan

[7] Department of Psychology, Graduate School of Humanities, Kobe University, Kobe, Japan

***Correspondence author :** Tatsuya Uebi, tuebi@emerald.kobe-u.ac.jp

No	Compound	Average composition (%)	
		Natural odor	Artificial odor
25	Nonanal	30.7	31.5
32	Dodecane	12.1	12.5
17	6-Methyl-5-hepten-2-one	7.4	7.6
20	Limonene	7.3	7.5
13	Heptanal	6.9	7.1
15	Benzaldehyde	5.6	5.8
27	Octanoic acid	5.3	5.4
30	Decanal	4.7	4.8
12	Cyclohexanone	3.6	3.7
29	Menthol	3.3	3.4
19	Octanal	3.2	3.3
14	sec-Butyl formate	2.6	0.0
11	2(5H)-Furanone	2.6	2.7
23	1-Octanol	2.4	2.5
35	Nonanoic acid	2.2	2.2

Supplemental Table S1. Mean values for the composition of natural head odor of a newborn baby immediately after birth compared with that of an artificial mixture mimicking a baby head odor. Percentages of the odor compounds used to concoct the artificial odor were calculated based on their mean content for the 15 most-abundant volatile substances in the odors of Babies 1 and 2. Numbering for the compounds corresponds to that for the compounds listed in Table 1. Compound No. 14 (highlighted) could not be purchased commercially and so could not be included in the artificial odor.

No	Compound	Average composition (%)	
		Natural odor	Artificial odor
25	Nonanal	33.1	34.3
32	Dodecane	12.6	13.0
16	Hexanoic acid	9.0	9.3
27	Octanoic acid	8.8	9.1
35	Nonanoic acid	7.9	8.2
30	Decanal	4.7	4.8
17	6-Methyl-5-hepten-2-one	3.8	3.9
13	Heptanal	3.7	3.8
15	Benzaldehyde	3.1	3.2
19	Octanal	2.8	2.9
37	3-Hexyn-1-ol	2.7	2.8
23	1-Octanol	2.4	2.5
29	Menthol	2.0	2.1
4	2,4-Pentadienal/Hexanal	1.8	0.0
34	4-Methyldecane	1.8	0.0

Supplemental Table S2. Mean composition of the 15 most-abundant components in natural and artificial head odor of a newborn baby 2-3 days after birth. Percentages for the artificial odor were calculated as in Supplemental Table 1, using the results from analysis of compounds in the head odor of Babies 3-5. Numbering for the compounds corresponds to that for the compounds listed in Table 1. Compound No. 4 is estimated to be 2,4-pentadienal or hexanal but identification requires further investigation, and Compound No. 34 could not be commercially purchased, so these two highlighted compounds were omitted from the artificial odor product.

No	Compound	Average composition (%)	
		Natural odor	Artificial odor
6	2,4-Dimethylheptane	13.8	16.8
3	4-Methylheptane	13.0	15.8
10	3-Ethylhexane	12.7	0.0
16	Hexanoic acid	11.9	14.4
8	2,4-Dimethyl-1-heptene	11.8	14.3
27	Octanoic acid	9.1	11.1
5	4-Methyl-1-pentanol	6.8	8.3
25	Nonanal	5.5	6.7
35	Nonanoic acid	4.6	5.6
12	Cyclohexanone	4.4	5.3
21	Heptanoic acid	1.5	1.8
33	2-Methyl-2-decanol	1.5	0.0
4	2,4-Pentadienal/Hexanal	1.5	0.0
26	Benzoic acid	1.0	0.0
2	2-Butenoic acid	0.7	0.0

Supplemental Table S3. Mean odor composition of the 15 components most abundant in the natural or artificial odor pattern of amniotic fluid. Percentages of the odor compounds used were calculated based on their mean amounts in the odors of Moms 1 and 2. Numbering for the compounds corresponds to the numbers in Table 1. Compounds omitted from the artificial odor products are highlighted: the identity of compound No 4 requires further investigation (cf. Suppl. Table S2); the amounts of Compound no. 26 are negligible (at the limit of detection; and the remainder were not commercially available.
